# Ultra-Processed Food, Reward System and Childhood Obesity

**DOI:** 10.3390/children10050804

**Published:** 2023-04-29

**Authors:** Valeria Calcaterra, Hellas Cena, Virginia Rossi, Sara Santero, Alice Bianchi, Gianvincenzo Zuccotti

**Affiliations:** 1 Department of Internal Medicine and Therapeutics, University of Pavia, 27100 Pavia, Italy; 2 Pediatric Department, Buzzi Children’s Hospital, 20154 Milano, Italy; 3 Laboratory of Dietetics and Clinical Nutrition, Department of Public Health, Experimental and Forensic Medicine, University of Pavia, 27100 Pavia, Italy; 4 Clinical Nutrition Unit, General Medicine, Istituti Clinici Salvatore Maugeri Istituto di Ricovero e Cura a Carattere Sscientifico, 27100 Pavia, Italy; 5 Department of Biomedical and Clinical Science, University of Milano, 20157 Milano, Italy

**Keywords:** obesity, adolescents, children, ultra-processed food, nutrients intake, unhealthy dietary pattern, feeding regulation, brain reward system

## Abstract

Obesity and overweight are a major public health problem globally. Diet quality is critical for proper child development, and an unhealthy diet is a preventable risk factor for noncommunicable diseases (NCDs), such as obesity. Consumption of sugar-sweetened beverages and ultra-processed foods (UPFs) in childhood may increase the BMI/BMI z-score, body fat percentage, or likelihood of overweight. A strict feeding regulation system allows for sufficient food to be consumed to meet ongoing metabolic demands while avoiding overconsumption. This narrative review explores the issues of obesity and the regulation of food intake related to reward systems and UPF consumption. Nutrient composition alone cannot explain the influence of UPFs on the risk of obesity. Furthermore, the non-nutritional properties of UPFs may explain the mechanisms underlying the relationship with obesity and NCDs. UPFs are designed to be highly palatable, appealing, and energy dense with a unique combination of the main taste enhancer ingredients to generate a strong rewarding stimulus and influence the circuits related to feeding facilitation. How individual UPF ingredients influence eating behavior and reward processes remains not fully elucidated. To increase the knowledge on the relationship between UPFs and pediatric obesity, it may be useful to limit the rapid growth in the prevalence of obesity and subsequent related complications, and to develop new strategies for appropriate food and nutrition policies.

## 1. Introduction

Obesity and overweight are major public health problems globally [1]. There are many underlying causes, with genetic predisposition and environmental factors undoubtedly together promoting this clinical condition [2].

Consumption of ultra-processed foods (UPFs), i.e., ready-to-eat foods derived from substances extracted or refined from whole foods, with flavorings, colors, and other cosmetic additives added, with few, if any, whole foods remaining [3] has increased overall in all socioeconomic classes [4,5]. However, UPF consumption possesses a risk of malnutrition, particularly in socioeconomically disadvantaged groups or in low- and middle-income urban communities [6]. Children and adolescents are also consuming increasing amounts of UPFs, and these foods can have numerous health consequences. 

In particular, diet quality is critical for proper child development, and an unhealthy diet is a preventable risk factor for noncommunicable diseases (NCDs), such as obesity [5,7]. In fact, the consumption of sugar-sweetened beverages (SSBs) and UPFs in childhood may increase the BMI/BMI z-score, body fat percentage, or likelihood of overweight/obesity [5,8]. In addition, exposure to sugary foods in early childhood may result in a dietary preference for sweet foods in adulthood, limiting the dietary intake of healthy foods [5,6]. 

A very strict feeding regulation system allows for sufficient food to be consumed to meet the ongoing metabolic demands while avoiding overconsumption [9,10]. It is based on vagus nerve signals, metabolic signals (i.e, the blood glucose concentration), and neuroendocrine signals, determined by different hormones, such as ghrelin, intestinal peptide hormones (GLP-1, cholecystokinin, and PYY), insulin, and others, as described in Section 4.1. However, studies have shown that circuits related to feeding facilitation are linked to the reward system [11,12,13].

The objective of this review is to explore the relationship between the consumption of UPFs and the development of pediatric obesity, with a focus on the role of reward systems in regulating food intake.

This narrative review explores the issues of obesity and the regulation of food intake related to reward systems and UPF consumption. The diet consumed during childhood is strongly indicative of future eating habits. To increase the knowledge on the relationship between UPFs and pediatric obesity, it may be useful to limit the rapid growth in the prevalence of obesity and subsequent related complications and to develop new strategies for appropriate food and nutrition policies.

## 2. Methods

**Figure 1:**
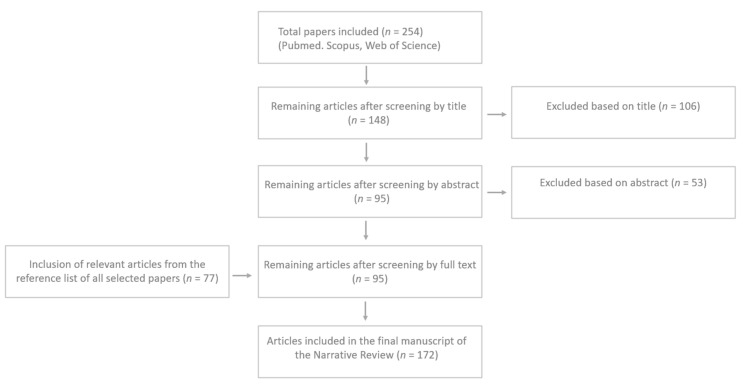
Graphical representation showing the process of the paper selection and exclusion used in writing this narrative review.

We developed a narrative review of the literature [14,15] on the topic of UPFs, reward systems, and childhood obesity. Articles in the English language; original observational studies, guidelines, consensus position statements and commentaries, systematic reviews, meta-analyses, and reviews published on a specific topic within a predetermined time range (2000–January 2023) were considered. In the chapter on feeding regulation and the brain reward system, the experimental studies were also included given the peculiar topic of the neurological discussion on the reward system. Starting from a total of 254 papers, the authors assessed the abstracts (*n* = 148) and reviewed the full texts of the relevant articles (*n* = 95) analyzed in order to provide a critical discussion. Additionally, the reference list of all articles was checked to identify the relevant studies (*n* = 77); a total of 172 papers were finally included. In Figure 1, the process of paper selection and exclusion is shown. 

The research terms adopted (alone and/or combined) were obesity, adolescents, children, ultra-processed food, nutrients intake, unhealthy dietary pattern, feeding regulation, brain reward system. The PubMed, Scopus, and Web of Science databases were used for research purposes. The contributions were independently collected by V.R., S.S., and A.B. and critically analyzed and discussed with V.C. and H.C. The resulting draft was critically revised by V.C., H.C., and G.Z. The final version was approved by all. 

## 3. Childhood Obesity

Obesity and overweight, defined by the World Health Organization (WHO) as abnormal or excessive fat accumulation constituting a health risk, represent a major public health problem, affecting all age groups of the population. They have led to an increase in both direct and indirect health care costs [1,2,16]. Epidemiological studies state that obesity prevalence has tripled over the past four decades globally [1,16,17]; in the United States, up to one third of children and adolescents are overweight or obese [18], and Italy is one of the top countries for a prevalence of obesity and overweight in pediatric-aged groups [19]. Overall, the prevalence of obesity has increased dramatically in children and adolescents, rising from 0.7 percent to 5.6 percent in boys, and from 0.9 percent to 7.8 percent in girls between 1975 and 2016 [20,21]. Data show that the most rapid weight gain occurs between the ages of 2 and 6 years, and 90% of children who were obese at 3 years old were also overweight or obese during adolescence [20,22]. However, since the early 2000s, in some high-income countries, such as France, Norway, Denmark, Sweden, the United States, Japan, and Australia, rates of childhood overweight and obesity appear to be declining or at least stabilizing [21,23]. However, since the data on childhood obesity in these countries indicate that the number of cases remains stable, it is assumed that the incidence of new cases of obesity is still high [20]. Obesity prevalence is related to the interaction of many factors, including biological, genetic, socioeconomic, ethnic, and social factors [24]. An obesogenic environment acts at several levels: familial (e.g., practice of physical activity, dietary habits, sleep-time, and screen-time), local community (e.g., child care, schools, parks, public transports), and sociopolitical (e.g., food industry and marketing, transportation systems, agricultural policies, subsidies) [20,25,26,27]. Dietary factors contributing to the obesity risk in children and adolescents include the excessive consumption of energy-dense, micronutrient-poor foods, such as UPFs [25,27,28]. High screen time also influences the risk of obesity in children and adolescents at several levels, as it leads to the increased exposure to food marketing, meals with little focus on what is being eaten, increased sedentary lifestyle, and reduced sleep time [20,25,27,29]. It has been observed how, in children, the increased intake of energy-dense foods and beverages occurs during or shortly after exposure to advertising; therefore, the two are related [30]. Poorolajala et al. [31] conducted a systematic review and meta-analysis regarding the behavioral factors capable of influencing childhood obesity. Their work showed that sufficient physical activity (at least 60 min of moderate- to vigorous-intensity physical activity per day, or 300 min per week), eating breakfast every day, and eating sweets ≥ 3 times/week have a significant effect in reducing the risk of childhood obesity [31]. In particular, breakfast consumption and physical activity were the first and second most powerful protective factors against excess weight gain in children and adolescents. In contrast, insufficient fruit and vegetable consumption (≤4 times a day or ≤5 times a week) and snack consumption (≥4 times a week) had a nonsignificant effect on childhood weight loss [31].

Moreover, breastfeeding for a short duration (<4 months), insufficient sleep (less than at least 9–12 h/day for children aged 6–12 years or 8–10 h/day for children/adolescents aged 13–18 years), watching too much TV (>1–2 h/day), consuming SSBs (≥4 times/week), and smoking had a significant effect on childhood weight gain [31]. Watching too much TV and a lack of sufficient sleep were the first and second most powerful risk factors of childhood overweight and obesity. In contrast, playing computer games (>2 h/day), eating fast food (≥3 times/week), fried foods (≥3 times/week), and drinking alcohol had no significant effect on childhood weight gain [31].

As stated by Kavey et al. [32], almost 40 percent of the total energy consumed by young people aged 2 to 18 years is empty calories. In particular, SSBs consist exclusively of empty calories and, according to Kavey et al., represent the main source of added sugars in children’s diets [32]. High SSB consumption has been associated with an increased BMI, waist circumference, and body fat percentage among children and adolescents. Discussing the Italian data on the consumption of SSBs, it has been observed that 36% of children aged 8–9 years participating in Okkio alla Salute, the Italian arm of the Childhood Obesity Survey Initiative, consumed them at least once a day [33]. As also shown by Rousham et al. [5], SSB consumption can increase the BMI, body fat percentage, and risk of overweight/obesity [34]. In addition, the high consumption of SSB is associated with numerous cardiovascular risk factors, both independently and as a result of obesity [32,35]. These risk factors in childhood are associated with accelerated atherosclerosis and early cardiovascular disease [32]. Therefore, reducing the consumption of SSB should be considered a critical dietary approach for cardiovascular risk reduction from early childhood.

Furthermore, childhood obesity prevalence is known to be higher in Hispanic and African American populations [36,37,38], but it is increasing in both low-income and high-income countries [39]. Certainly, the COVID-19 pandemic has played an important role in its rise, due to social isolation and sedentary lifestyles [40,41]. Other factors contributing to the obesity epidemic include a low birth weight for the gestational age (small for the gestational age, SGA), formula feeding, excessive protein intake during childhood [18,42,43], epigenetics, and altered gut microbiota [44,45].

Specifically, the BMI (weight/height^2^; kg/m^2^) is an indirect measure of fat mass in children and adolescents over 2 years of age [46], and is typically interpreted using percentiles based on age and sex. Between 0 and 2 years of age, the weight/length ratio is preferred. In most individuals, the BMI is believed to be an accurate parameter for estimating overweight and obesity, although it is less reliable in individuals with a well-developed lean mass, where a high BMI does not depend on an increased fat mass. In this case, a body composition analysis is useful and more appropriate by means of different tools, such as skin fold thickness, bioelectrical impedance, hydrodensitometry, dual-energy X-ray absorptiometry (DEXA), and air displacement plethysmography [2].

At present, there are three main classifications of pediatric obesity: the U.S. Centers for Disease Control (CDC), International Obesity Task Force (IOTF), and WHO [47]. These classifications involve the collection of anthropometric measurements (i.e., weight, height, circumference) to be reported in sex- and age-specific growth curves, allowing for the assessment of the presence of overweight or obesity.

The U.S. CDC growth charts can be used for children aged 2 to 20 years; according to the CDC, obesity is defined by a BMI ≥ 95 percentile [46,48]. The IOFT considers the obesity cut-off as related to gender-specific BMI charts, and is evaluated using large databases from different countries [47,49]. The WHO defines overweight as having a BMI ≥ 1 SD and obesity as having a BMI ≥ 2 SD above the WHO child growth standard median for children aged 5 to 19 years, using the 2007 WHO charts [46,50]. For children under than 5 years of age, the WHO defines overweight as having a weight-for-height >2 SD and obesity as 3 SD above the WHO child growth standard median, using the 2006 WHO charts [50]. The CDC, IOFT, and WHO growth charts are based on different databases and have different cut-offs for defining overweight and obesity, but they all provide useful tools for health care professionals to evaluate and monitor children’s growth and development.

Obesity predisposes pediatric patients to various inflammatory, metabolic, and endocrine dysfunctions, as well as various complications and comorbidities that can affect almost any system [48,51,52]. Changes in the immune system are secondary to the pattern of low-grade systemic chronic inflammation (SLGCI) that are typical of obesity, and are characterized by altered circulating levels of acute phase reactants and cytokines [53,54]. In childhood, obesity can lead to a range of comorbidities, including insulin resistance, metabolic syndrome, hypertension, sleep apnea, asthma, and non-alcoholic fatty liver disease (NAFLD). Children who are obese are also at increased risk for developing type 2 diabetes, cardiovascular disease, and joint problems later in life [43,47,55]. Endocrinologically, there is an increased risk of developing precocious puberty in children with obesity and menstrual irregularities in adolescent girls [56,57]. Furthermore, it must not be forgotten that sleep disorders, such as obstructive sleep apnea (OSA) [18,58], as well as psychological problems, such as depression, anxiety, low self-esteem, body image and peer relationships, and eating disorders are associated with obesity. [59,60].

Therefore, it is paramount to intervene early in childhood to prevent and/or treat obesity and its associated complications. First-line approaches are based on lifestyle and behavioral changes. Lifestyle and behavioral changes in the individual are focused on increasing daily exercise and improving the diet quality by limiting excessive energy intake and poor diet [18,61,62]. Changes in the community environment, including food security, promoting sustainable healthy food choices through taxes on unhealthy products, such as UPFs [63], bans on advertising unhealthy snacks, in addition to daily physical activity in schools and childcare centers, as well as easy access to playgrounds and green spaces, all lead to a decrease in sedentarism [62]. In secondary and tertiary prevention along with those first-line interventions, pharmacotherapy and/or bariatric surgery may be considered [47].

## 4. Feeding Regulation and Brain Reward System

Strict feeding regulation is necessary for all animals in order to maintain an adequate energy balance. The existence of a very precise feeding regulation system allows for a fine regulation of food intake that is adequate to fulfill metabolic demands and to avoid overconsumption, that leads to a positive energy balance, resulting in body fat accumulation [64,65]. 

A dysfunction of the neural circuits controlling eating behaviors may lead to pathological conditions, such as hypophagia or hyperphagia. The former condition may result in restrictive eating behaviors while the latter may induce excessive weight gain. Moreover, the neurocircuits involved in feeding control, previously fully functioning, may be compromised by later pathological conditions, such as substance abuse. Medication and drug abuse, in fact, act on the reward system and may alter feeding regulation, suggesting an overlap between reward and feeding circuits within the brain [66,67]. 

Circuits most closely related to feeding facilitation are linked with those most closely related to reward-driven behavior. A detailed review of these systems will facilitate our understanding of pathologies that rely on feeding and reward circuits [66].

Although it was already known that feeding and reward circuits were closely related, these two topics have often been investigated separately. For example, several studies have focused on the role of specific brain regions involved in the regulation of body weight, food intake, and energy expenditure [68,69,70,71], while others have examined the role of neural circuits involved in reward-driven behavior [72,73,74], but relatively few have considered the two together [75,76]. 

To simplify, feeding behavior can be distinguished into homeostatic feeding, that is, the pathway that increases the motivation to eat to make up for depletion of energy reserves, and hedonic feeding is the pathway that increases the desire to consume highly palatable foods, even though they are not necessary for the body’s energy balance [65]. The homeostatic and hedonic feeding systems are both activated during all feeding situations. The degree of activation of each depends on different elements, such as food consumption and/or the physiological state of individuals.

### 4.1. Feeding Regulation

Both hunger and satiety are biological processes that were first described more than 20 years ago [77], and later discussed in several reviews [9,78]. They rely on physiological events that control appetite, as well as on psychological experiences related to the process of eating. The eating process is generally driven by the ‘hunger signal’, while its cessation depends on different signals that are generated during food intake [10].

Moreover, the mechanism of hunger [79] depends mainly on three crucial signals: the electrical signal, i.e., embodied by the vagus nerve, that detects the emptiness (or fullness) of the stomach; the metabolic signal, determined by the blood glucose concentration (e.g., hypoglycemia); and the neuroendocrine signal, i.e., the secretion of the ghrelin hormone from the P cells, located in the gastric fundus.

Satiety, moreover, is the process that leads to meal cessation and determines the dietary intake. The first signal regarding food consumption comes from the stomach, that provides feedback based on the stretching/distension state of the walls and the level of osmotic load. Medium-term satiety is metabolically controlled by intestinal peptide hormones, including GLP-1, cholecystokinin (CCK), and PYY, that are released when food passes through the gastrointestinal tract and play a major inhibitory role on food intake [80]. Additionally, long-term satiety is regulated by the concentrations of insulin, glucose, as well as plasmatic amino acid concentrations and hepatic nutrient oxidation levels. 

However, the homeostatic control of food intake can be influenced by the “stress system” [81]. Stress acts as a destabilizing factor that can increase or decrease food intake. By its nature, the response to stress suppresses appetite: when homeostasis is threatened, the sense of hunger, the search for food, and the digestive activity are inhibited because they are not a priority. Stress hormones, adrenaline, and cortisol contribute to reduce blood glucose levels by stimulating insulin secretion. High insulin levels in the blood act as a signal of satiety. Moreover, glucocorticoids (GCs) are able to stimulate appetite. Firstly, cortisol promotes the negative feedback on the CRH secretion, thereby reducing the anorexic signal. In addition, high levels of cortisol increase the production of ghrelin, that stimulates hunger. Cortisol has a longer half-life in the blood than CRH, and exerts long-term effects through the interaction with specific intracellular receptors. This mechanism allows to recover the energy spent during the stressful events, according to a perfect homeostatic mechanism. Moreover, in the case of chronic stress, GC levels are kept chronically high, leading to increased feeding and, consequently, obesity. In more detail, GCs seem to stimulate the appetite for very palatable and high-calorie food. Together, “satisfying food” and cortisol directly activate the dopamine reward circuits. In addition, while promoting leptin release from adipose tissue, GCs decrease the hypothalamus sensitivity to the hormone, contributing to leptin resistance, thus reducing the satiating action. Leptin also inhibits the NAc, the area of the brain involved in cognitive reward processes; therefore, a vicious circle leads to a constant increase in the intake of “comfortable” food to maintain the pleasure/reward effect.

Such as leptin, GCs also stimulate insulin secretion from the pancreas, which normally reduces both food intake and reward circuits. However, elevated chronic levels of GCs contribute to insulin resistance [82]. Several studies in children have observed relationships between stress and unhealthy dietary practices, including increased snacking [83], and elevated risk for problems with weight during adolescence and adulthood [84]. In a controlled study of 9-year-olds, children who felt more stressed by school challenges tended to eat more comfort food [85].

The link between food intake and reward circuits, as well as sensory and cognitive processes may also lead to meal anticipation, altering the overall quality and quantity of the meal. Hence, the brain sums up all of the signals from the various processes involved in appetite control, integrating hedonic and homeostatic appetite control, with signals related to sensory and metabolic satiety. Particularly in modern Western cultures, palatable, calorie-dense foods are widely available. This obesogenic food environment determines an explicit or implicit awareness of palatable foods, that induces the so-called hedonic (or pleasure-based) hunger [86]. Many factors may contribute to stimulating hedonic hunger, such as food advertisements, smelling food, negative moods, and seeing others eat. It leads to a superfluous consumption of highly rewarding, energy-dense foods with a consequent inevitable weight gain. Moreover, the repeated consumption of highly palatable foods in heterogeneous environments might determine the motivational salience to diverse situations [86]. In other words, the modern obesogenic food environment can promote on its own eating-related thoughts and desires. 

### 4.2. Brain Reward System

**Figure 2:**
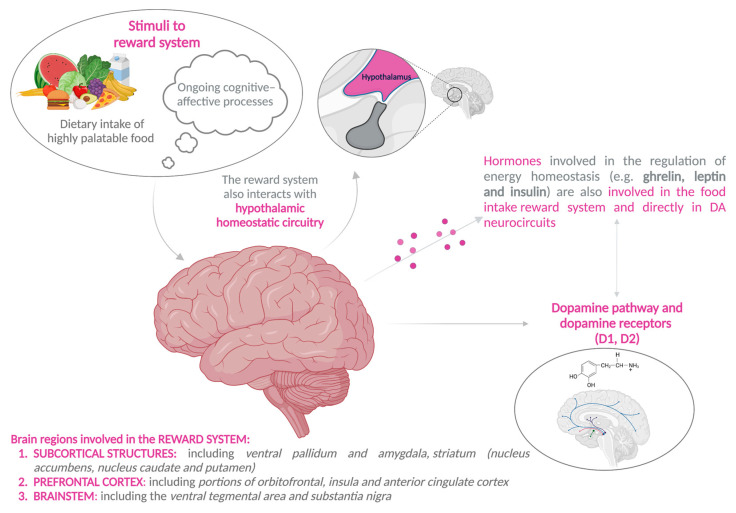
Graphical representation of the reward system. In the reward system, subcortical structures, prefrontal cortex, brainstem, and hypothalamic circuity are involved.

The neural circuitries belonging to the reward system consist of (i) subcortical structures, including ventral pallidum and amygdala, striatum (nucleus accumbens, nucleus caudate, and putamen), (ii) the prefrontal cortex, including portions of orbitofrontal, insula, and anterior cingulate cortex, and (iii) the brainstem, including the ventral tegmental area and substantia nigra [11,12,13]. The reward system also interacts with hypothalamic homeostatic circuitry, allowing relevant physiological hunger and satiety states to modulate the search for food rewards [13] or overriding the basic satiation signals generated in homeostatic centers [87], as shown in Figure 2. 

The reward system is stimulated by the dietary intake of highly palatable food, such as ultra-processed food, either through the activation of exteroceptive pathways, by anticipatory (visual and olfactory) and contextual (gustatory) stimuli to the consumption of ultra-processed food itself, or the interoceptive pathways by satiety signals (including gastric distension and satiety hormones produced by the gastrointestinal tract in response to the presence/absence of nutrients) [11]. In addition, the reward system is deeply influenced by the ongoing cognitive–affective processes, that ultimately determine the reward properties and affective value of the food, affecting appetite and eating behaviors [11]. 

**Figure 3:**
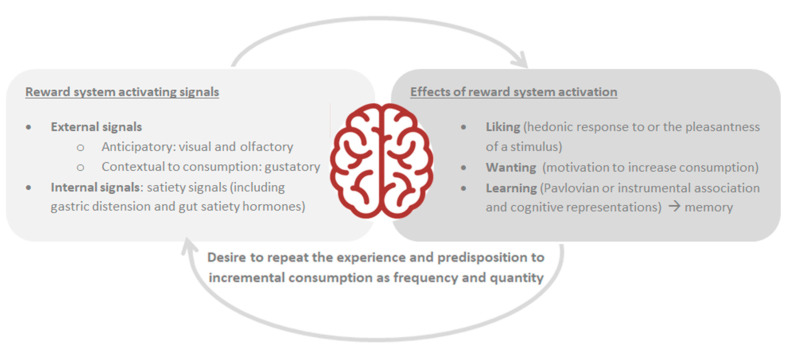
Graphical representation of the reward-behavior cycle. The reward system is activated by external or internal stimuli. The effects of the reward system activation involves several physiological processes: pleasure, wish, and learning, leading to a reward-behavior cycle with an escalation in the consumption of those unhealthy foods.

The rewards individually perceived involve several physiological components, including pleasure (hedonic response to or the pleasantness of a stimulus), wish (motivation to increase consumption), and learning (Pavlovian or instrumental association and cognitive representations), leading to a reward-behavior cycle [12,87] (Figure 3). 

In particular, while learning processes happen throughout the reward-behavior cycle, the pleasure processes tend to dominate the initial appetitive phase and the liking processes to dominate the subsequent consummatory phase that may lead to satiety [12]. 

Pleasure serves as an adaptive function, motivating individuals to pursue rewards necessary for performance, and thus playing a crucial role in human evolution; yet, in modern environments, the abundance of pleasure induces maladaptive pursuits, such as food overconsumption and binge eating episodes [12].

In fact, the reward circuitry is interestingly activated by both drugs and UPFs, triggering in both cases an escalation in consumption (“abuse” of ultra-processed foods) and subsequently making it more difficult for some individuals to quit or reduce consumption [11,70]. There are, however, significant differences between drugs and food consumption, dopaminergic signaling elicited by drugs remains active in the long term, whereas it does not happen with palatable food intake [11,87]. 

Focusing on wish, the UPFs may stimulate appetite even when energy requirement has been satisfied, thus overcoming homeostatic hunger/satiety action mechanisms [87,88]. Over time, as a result of repeated exposure to UPFs, ‘wish’ selectively becomes higher, especially if additional predisposing factors, such as stress and negative emotions, are co-present, favoring impulsive comfort food overconsumption leading to unhealthy dietary choices and weight gain [11,13,88]. 

Considering the learning process, the reward system will remind the individual of the pleasant sensations linked to UPF consumption, and will try to repeat the experience every time, whenever there will be an opportunity [12,88].

The reward system recognizes the involvement of neurotransmitters, especially dopamine [11,88], and neuropeptides, including endogenous opioids [11].

Focusing on dopamine, as stated before, food’s ability to activate the mesolimbic dopamine (DA) system has been demonstrated. Food, by promoting the rapid activation of DA neurons, encourages behaviors directed toward reward acquisition [89]. However, it is still difficult to delineate exactly the role of the DA system and receptor subtypes in food reward.

Dopamine is the crucial catecholamine neurotransmitter synthesized by mesencephalic neurons in the substantia nigra (SN) and ventral tegmental area (VTA). DA neurons originate in those nuclei and project to the striatum, cortex, limbic system, and hypothalamus, promoting control of coordinated movements and hormone secretion, as well as motivated and emotional behaviors [90,91]. Dopamine interacts with membrane receptors, that can be classified into two groups based on their structural and pharmacological properties: the D1-like and the D2-like receptors. D1 receptors are localized post-synaptically; whereas D2 receptors are localized pre-synaptically and have the function to reduce neuronal excitability, decreasing DA synthesis, packaging [92], and release [93,94]. 

A previous study involving rats showed that the knockdown of the striatal dopamine D2 receptor by lentivirus-mediated short hairpin interfering RNA rapidly induced addiction-like reward deficits and compulsion-like food seeking [95]. Because of the reduced D2 receptors’ density, the dorsal striatum is less responsive to food reward compared with lean control groups in rats and obese humans.

Several human studies point out that subjects with obesity and drug addicts tend to show a reduced expression of D2 receptors in striatal areas. Positron emission tomography (PET) studies suggest that the availability of D2 receptors decreases in individuals with obesity in proportion to their BMI [96]. 

Another study that used functional magnetic resonance imaging (fMRI), found that some individuals eat more to compensate for a hypofunction of the dorsal striatum, particularly those with a genetic polymorphism in the D2 receptor gene (DRD2/ANKK1), which is associated with lower striatal D2 receptor density [97]. 

These findings underscore that individuals who show reduced striatal activation during food intake have an increased risk of obesity, particularly those with genetic alterations in DA signaling. Thus, it is possible that, in individuals with obesity, as in chronic drug abusers, there are low basal DA concentrations and exaggerated periodic DA release related to food intake (or drug abuse), in association with the low expression or dysfunction of D2 receptors [98].

Moreover, it has been found that a high-fat diet (HFD) attenuates dopamine D2 receptor signals in the striatum, resulting in hedonic overexposure. Kozuka and colleagues [99] reported that γ-orizanol, a bioactive component present in rice, attenuates the preference for HFD through hypothalamic control. They hypothesized that γ-orizanol can also modulate the functioning of the reward system of the brain. In the striatum of mice fed a HFD, the production of D2 receptors was decreased due to an increase in DNA methylation of the promoter region of the D2 receptors. Oral administration of γ-oryzanol seems to decrease the expression and activity of DNA methyltransferases (DNMTs), thereby restoring the level of D2Rs in the striatum. The authors conclude that γ-orizanol is an epigenetic modulator and it may be a particularly promising anti-obesity substance.

Not only striatal areas, but also other brain areas are probably involved in dopamine neurocircuitry. 

Recent findings reveal that hormones involved in the regulation of energy homeostasis, such as ghrelin, leptin, and insulin, are also involved in the food intake reward system and directly in DA neurocircuits [67,89].

DA neurons in the VTA express receptors for leptin, a hormone produced and secreted by adipose tissue, that can promote a downregulation of DA neurons [100]. 

Evidence shows that the direct administration of leptin in the VTA induces a decrease in food intake, suggesting that leptin signaling in the VTA normally suppresses DA pathways, and consequently is able to decrease food intake. Human studies also show that leptin can control reward responses [101,102]. Furthermore, studies have shown that in a leptin-deficient condition, images of palatable foods generate a greater craving response, even when the subject has just been fed, whereas after leptin treatment, images of palatable foods generate this response only in the fasting state [101,102]. Leptin reduces NAc and mesolimbic activation, decreasing rewarding responses to food by acting on the DA system.

Conversely, the peptide hormone ghrelin, that is produced in the stomach and pancreas, is known to increase appetite and food intake. Ghrelin receptors are located in hypothalamic centers and the VTA, where they can stimulate an increase in DA neuronal activity, promoting appetite [103].

Additionally, insulin, involved in controlling glucose metabolism and inhibiting feeding, also plays a role in regulating the DA system in the brain [104]. Insulin receptors are strongly and widely expressed in different brain regions, such as the striatum and midbrain. As reported, a direct administration of insulin into the VTA reduces food intake and represses the feeding of a high-fat sugary diet under conditions of satiety [105,106]. Interestingly, deletion of the insulin signaling in the catecholaminergic neurons resulted in increased sucrose sensitivity, promoting an obese phenotype [107]. Insulin increased the dopamine re-uptake transporter (DAT) mRNA levels, leading to the enhanced clearance of dopamine from the synapse, and therefore reducing DA signaling [64].

However, nowadays the assumption that dopamine is not the main neurotransmitter involved in the process of “pleasure” is gradually emerging [12,13]. In fact, evidence has emerged that dopamine loss does not necessarily reduce pleasure. In humans, Parkinson’s patients see their dopamine levels depleted due to their disease, yet they still manage to experience normal sensory pleasure, for example when savoring a sweet taste [108,109].

However, other neurotransmitter systems, e.g., the endocannabinoid system and GABA-ergic neurotransmitters, are also involved in the process of food liking, acting in specific forebrain limbic structures or “hedonic hotspots”, including the medial NAc shell and the posterior ventral pallidum [11,12].

While there are similarities between the reward system in adults and children, there are also important differences. One of the main differences between the reward system in adults and children is the way that it responds to rewards [110]. Studies have shown that children’s and adolescents’ reward systems are more sensitive to rewards than adults’ reward systems [111]. This means that children may experience greater pleasure and motivation from rewards, such as food. Another difference between the reward system in adults and children is the way that it develops over time [112]. The reward system is not fully mature at birth and undergoes significant changes during childhood and adolescence. For example, the prefrontal cortex continues to develop well into early adulthood [113]. As a result, children and adolescents may be more prone to impulsive behavior and risk-taking, that can affect the way their reward system responds to stimuli [114]. In summary, while there are similarities between the reward system in adults and children, there are also important differences in terms of sensitivity to rewards and the way it develops over time. Understanding these differences can help us to better understand how the reward system influences behavior and motivation in different age groups.

## 5. Ultra Processed Food in Childhood Obesity

There are three main classifications of processed food items. The Center for Epidemiological Studies in Health and Nutrition, School of Public Health, University of São Paulo, Brazil has produced the NOVA classification [3], which groups food into four subgroups based on the extent and purpose of industrial food processing, without providing any indication of the nutritional content of foods [115,116]. Therefore, transformation of foods into substances, the chemical modification of substances, and use of additives aims to create products that are highly profitable (cheap ingredients, long shelf life), convenient (ready-to-eat), and hyper-palatable [3]. NOVA system [115] distinguishes four main food subsets: unprocessed or minimally processed foods (in which salt, sugar, oil, and other substances are not added), processed culinary ingredients (derived from the previously described group and processed, such as pressing, refining, grinding, milling, and drying, processed foods (added with salt, sugar, and other substances in order to make unprocessed food more palatable), and ultra-processed food (usually derived from a range of industrial techniques and processes).

The European Prospective Investigation into Cancer and Nutrition [117] proposed three main UPF categories: highly processed, moderately processed, and unprocessed foods. 

Furthermore, Siga classification classifies foods based on its processing; combining the four NOVA groups with four other new reductionist subgroups that consider the impact of processing on the food/ingredient matrix, the content of added salt, sugar and fat, nature and number of ultra-processing markers, and levels of risky additives [116,118].

In addition, the European Food Safety Authority (EFSA) has developed a system for defining foods that combines both chemical exposure from food and the dietary assessment of food-intake [96], while the European consortium on food-composition data (Eurofir) has adopted the sophisticated LanguaL food coding system [110,111].

The NOVA classification is the one most widely used internationally in epidemiological studies; however, there is still an ambiguous food classification, especially in terms of the degree of processing and nutritional content [112].

Overall, UPFs are usually energy dense, high in free sugars, saturated fat, and sodium, and they are highly palatable, impacting the glycemic load. Moreover, they are low in protein, dietary fiber, micronutrients, and phytochemicals, compared to their unprocessed/minimally processed counterparts [113,114,119]. Examples of UPS are soft drinks, flavored dairy drinks, packaged snacks and ice cream, and ready meals. 

The consumption of UPFs, that perfectly address the public’s demand for palatable, inexpensive food items with a longer shelf-life [116] is rapidly and dramatically increasing globally in both high- and lower-income countries, due to the “nutrition transition” phenomenon [112,116,120,121]. Indeed, in the last 40 years, we are witnessing a shift from “traditional” eating patterns (respectful of local culture and culinary traditions) to a global Western diet pattern, affecting diet quality, with UPFs dominating the market and contributing 10–60% of the individual total energy intake (TEI) in the country [112,116,122]. Epidemiological data has become even more alarming when stratifying UPF consumption by age group, since consumers of UPFs are mainly children and adolescents [123,124]. 

According to statistics, in the United Kingdom, the majority of 7-year-old children consume diets that predominantly include UPFs (white bread, cookies, carbonated drinks, chips and carbonated drinks, potato chips) [125], while UPFs provide 65% of the energy intake in primary and secondary school children’s habitual dietary intake [126].

In Canada and the United States, data are also similar: UPFs provide more than 55% of the daily energy intake [125,126]. According to a study by Neri et al., carried out between 2009 and 2014, and who described U.S. preschool children’s dietary patterns, UPFs accounted for nearly 60% of the daily energy intake [125]. Children and adolescents consumed mainly pizzas, soft drinks, and fruit juices [125].

In low- and middle-income countries, UPF consumption is lower overall (18–35%), but young children are still early adopters and the largest target of consumers [114,127,128]. In Mediterranean countries, UPF consumption is more modest [112]. This evidence becomes even more alarming considering the rapid escalation trend that occurred in Italy in a decade: in fact, those recent data are double compared to the INHES cross-sectional survey conducted in 2010–2013, in which children and adolescents were reported to consume about 26% of the daily energy intake from UPFs [129].

Socioeconomic status is a discriminating factor in children’s and adolescents’ dietary patterns. In Europe, the children of parents with a lower education, who are younger or with lower economic standards are more likely to consume poorer and cheaper diets, with a higher UPF consumption [116,130]. The presence of older siblings or babysitters seems also to be a risk factor for dietary patterns rich in UPFs [130,131]. With regard to modern lifestyles, several aspects, including frequent snacking and eating away from home, especially for breakfast, poor sleep quality, and urban context, have been associated with the dramatic rise in the consumption of UPFs in children and adolescents [116,129,132,133]. Instead, unlike in adults, a clear association between screen time while eating and UPF consumption has not been clearly established in children and adolescents [129], probably due to underreporting.

In terms of biological factors, it is worth noting that, while the association between UPF consumption and obesity has been established in adults, difficulties have been encountered with children and adolescents [116,134]. A recent systematic review found that only longitudinal studies with a long follow-up (>4 years) could establish a positive association in this population group, therefore hypothesizing that a consistent intake of UPFs over time is needed to affect the nutritional status and body composition of children and adolescents, and that dietary habits may significantly vary over time in this age range [134]. Moreover, a confounding factor that could account for the difficulties encountered is the physiological increase in body tissues in childhood and adolescence, that results in increased energy expenditure and metabolic activity [134].

Considering that food preferences are influenced by maternal habits and choices during pregnancy, UPF consumption in this period critically impacts on infant food preferences, eating behaviors, and weight gain [133]. Therefore, the exposure to artificial and enhanced UPF flavors in utero increases through “flavor conditioning” the likelihood of postnatal UPF acceptance by the infant, at the expense of healthier food options [133]. The diet consumed for the first few months of life, particularly for the first 24 months, is strongly indicative of future eating habits [125,131]. Therefore, consuming UPFs from an early age can have awful consequences for the impressionable palate of toddlers, representing a strongly characterizing element of the future diet [125]. According to the fact that food choices at the weaning stage shape tastes, Birch and Doub [135] have shown that their consequences on children’s weight status are long lasting. If early experience includes exposure to certain types of foods and tastes, then they will be more likely to accept specific foods and tastes. In the case of the high consumption of UPFs, young children’s diets will probably continue to be dominated by sweet or salty foods that are easily accepted [125,131,135]. 

Regarding psychological and behavioral predisposing factors towards UPF consumption, they encompass both children (e.g., emotional eating) and parental factors (e.g., household UPF availability, parental role models, the misunderstanding of children’s hunger/satiety state, pressure to eat) [133,136,137]. Parents play a direct role in feeding their infants and children, providing foods to the table, serving as a direct meaningful role model in teaching them what, how, and when to eat [133], since growing children do not have full autonomy on food choice [136]. 

Children are more prone to overconsume UPFs if their parents tend in turn to do so, emphasizing the role of family-centered interventions to prevent and treat childhood obesity by both nutritional education and intervention, aimed at reducing environmental exposure to UPFs and increasing awareness on the importance to consume healthier foods [133,136]. 

Moreover, parents risk overfeeding their infants if they feed according to their own perception about when and how much is appropriate, without following children’s hunger/satiety cues through responsive feeding practices [133]. Furthermore, parents who engaged in more restriction and pressure to eat tend to lead their children to eat more UPFs [133]; in any case, it is still unclear whether this is a causal mechanism or a consequence (i.e., parents are using restraint as an attempt to modulate their children’s intake) [133]. Other psychological and behavioral factors towards UPF consumption investigated in adults (e.g., poor self-rated health status, depression, stress and/or neurosis) [129] could still impact indirectly on their children in terms of household accessibility to UPFs.

High levels of UPFs in the diet have been correlated with an increased risk of various food-related noncommunicable diseases (NCDs), both in adulthood and in pediatric/adolescent age [138]. 

Two studies conducted in Brazil on preschool to school-aged children with low socioeconomic status showed that intake of UPFs was positively associated with a higher serum lipid profile and waist circumference [8,139].

Numerous studies have also demonstrated the association between exposure to UPFs and overweight and obesity [138,140,141]. Pathogenetic mechanisms can be found both in the nutritional and non-nutritional properties of UPFs. First, UPFs themselves are, by definition, high-energy dense. Considering that the regulation of food intake depends mainly on the volume consumed rather than the calories ingested, eating these products may promote excessive energy intake [142,143]. In addition to the consequences from excessive energy intake, several studies have focused on the negative health effects of the poor nutritional quality of food, as UPFs are high in added sugars, sodium, and trans and saturated fats, and low in fiber and micronutrients [144,145,146]. High intake of added sugars has been independently associated with the risk of cardiovascular mortality [147]; similarly, high sodium intake has been associated with deaths from cardiovascular causes and an increased risk of certain cancers, such as stomach cancer [148]. Furthermore, the typically low fiber levels of UPFs need to be considered, as several studies have shown an inverse association between fiber consumption and risk of all-cause mortality, particularly mortality related to cardiovascular disease, coronary artery disease, and cancer (e.g., pancreatic and gastric cancer) [149,150]. Prospective studies also found that the higher intake of UPFs’ predicted a higher total cholesterol, LDL cholesterol, TAG and/or increased waist circumference in children [129]. In addition, in recent years, links are beginning to be drawn between certain industrial food additives (or clusters of additives) and gut microflora dysbiosis, that increase intestinal permeability and inflammation [151].

Emerging evidence suggests that nutrient composition alone cannot explain the influence of UPFs on the risk of obesity and NCDs [140]. Furthermore, the non-nutritional properties of UPFs may explain the mechanisms underlying the relationship with obesity and NCDs. UPFs are typically highly palatable, portion-packed in large sizes, and persuasively marketed. Such mechanisms may promote overconsumption [30,140,152]. In addition, these foods that tend to be ready-to-eat with minimal preparation, may alter eating patterns, promote snacking, rapid eating rates, and inattentive consumption influencing digestive and neural mechanisms involved in satiety [144,153]. 

## 6. Ultra-Processed Foods and Reward System in Children

### 6.1. Nutritional Factors Characterizing UPFs with a Potential Impact on the Reward System and Predisposing toward Overconsumption

**Figure 4:**
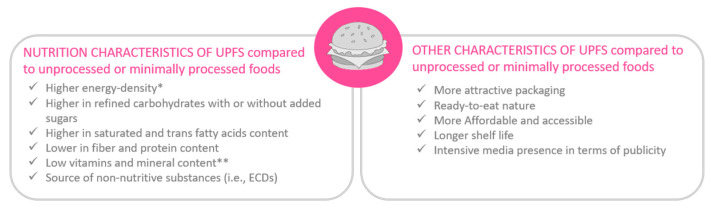
Schematic representation of nutritional and non-nutritional characteristics of ultra-processed foods compared to unprocessed or minimally processed foods. Exceptions to the statements made are indicated with an asterisk, respectively: * for light-soft drinks, ** for sodium.

UPFs share common nutritional characteristics (Figure 4), all indicating their poor nutritional value and justifying the consideration of UPFs as indicators of low quality nutrition [116,134]. 

Firstly, most UPFs are characterized by high energy density [134] (with the exception of diet cokes, sodas, and other acaloric beverages due to the use of non-nutritive additives), to the extent that they are the foods with the highest energy content per serving [154]. Thus, it appears that an excessive UPF consumption could cause the overconsumption of the daily energy requirement [155], leading to unhealthy weight gain. Additionally, the UPF consumption provides quickly available calories for the human body, due to the modified chemical and physical structure of the UPF matrix (e.g., by extensive milling), thereby simplifying and accelerating the processes of digestion and nutrient absorption [122,134]. These UPF features are also shared by SSBs, that provide energy in a liquid form, and are quickly available for the children’s body [34]. Focusing on the macronutrient content, UPFs are marked by the unnatural copresence of high refined carbohydrate levels, with or without added sugars [132], and saturated and trans fatty acid levels [134], all nutrients underlying the UPF reinforce the potential predisposition toward overconsumption [156]. It is noteworthy that the proportion of carbohydrates and fats derived from UPFs that are actually absorbed in the gut is very high given their low fiber content [134]. In addition, a lower protein content is reported in UPFs than in MPFs, potentially contributing, along with the low fiber content, to a less durable sense of satiety, and therefore promoting overeating and nibbling throughout the day [132,134,156,157]. The high glycemic load resulting from the intake of SSBs, leading to reduced satiety and satiation, thus represents another characteristic shared with UPFs [34].

In terms of micronutrient content, UPFs are often characterized by a high added sodium content intended to promote the high palatability of the finished product in combination with flavor enhancers; therefore, fostering UPFs’ rewarding nature [134,156]. Indeed, given their distinctive composition, UPFs have the potential to simultaneously stimulate different types of taste perceptions (sweet taste, salty taste, and/or fatty texture perception), aspect that may further drive the subject toward the excessive consumption of these food products. Regarding umami taste, umami ingredients (L-glutamic acid and its sodium salt, guanosine monophosphate, inosine monophosphate, and other ribonucleotides) are widely used in food production to enhance food flavor (savory) and to improve food consumption [158,159,160]. However, preliminary studies, including a Chinese study in humans, have shown a potential role of MSG in promoting the development of obesity [159]. In any case, more evidence is needed in humans, and especially studies in children, to verify the potential implications on this vulnerable population. In contrast, with regard to other micronutrients, UPFs are reported to be low in minerals, including potassium, zinc, and magnesium, and vitamins, including A, C, D, E, B3, and B12 [132,157]. Therefore, their predominant and frequent consumption in an individual’s dietary pattern can lead to the development of micronutrient deficiencies, which are particularly unfavorable in growing subjects, such as infants and children. 

Considering the food composition of UPFs as a whole, it is consequently evident how a stable and consistent consumption of UPFs, as part of children’s diet, leads inevitably to a nutritionally unbalanced eating pattern [112,132]. 

Additionally, due to the multitude of sequences of processes used to produce the final product [161], UPFs are also a source of exposure to non-nutritive substances, such as endocrine disruptors (ECD) (e.g., phthalates and bisphenol A) and neoformed contaminants (e.g., acrylamide and hetero-cyclic amines), respectively, due to packaging and high temperature heat treatments [129,134,151]. In addition, there are currently 13 NNSs (non-nutritive sweeteners or artificial sweeteners) approved for use globally for reducing the energy and sugar content, while still imparting sweetness to food products, such as carbonated beverages, fruit drinks, dairy products, and confectionery [160,162]. Nevertheless, evidence emerges in the literature of adverse health effects in humans, including in children, to the intake of food products containing NNSs, including alterations in microbiota composition, in the pancreatic post-prandial endocrine response, and in the cephalic mealtime response [163]. Combining this alarming preliminary evidence with the significant NNS exposure in early childhood, it is imperative to pursue more studies in this field to determine whether chronic NNS consumption throughout childhood leads to an increased risk of developing NCDs, potentially leading to changes in the recommendations of NNS use in the pediatric populations [163]. Other distinctive UPF aspects, as industrial products, are their attractive packaging, ready-to-eat nature, affordability, accessibility, longer shelf-life, and their intense media presence in terms of advertisement [122,133,154,164,165]. UPFs constitute in consumers’ perception, both time and money saving food options, since they require little to no culinary preparation, and they have convenient prices, due to large-scale production and low cost ingredients [133,157,164,165], a combination that is hard to resist. UPFs and PFs are generally easier food options to find when eating away from home than MPFs and UPFs, with larger portion sizes and a virtually limitless variety as part of a obesogenic environment [116,132,155,156]. There is also convenience for supermarkets to buy and resell UPFs, because of transport and storage easiness, and because of the high profit potential, due to lower prices in the market, enhanced palatability, and massive advertising campaigns [157,165]. In fact, UPFs are heavily marketed with aggressive and ubiquitous publicity [132,133,157,161], therefore their consumption is generally perceived as socially acceptable by the public [156]. 

In conclusion, considering all of these factors, it is thus not surprising that UPFs are currently dominating the food supply across the globe [133], also spreading to the emergent markets in developing countries, with an increased availability alongside access to supermarkets and fast-food chains [132]. 

### 6.2. Effect of UPFs on the Reward System, Promoting Excessive Energy Consumption

UPFs are in every respect comfort food: they are designed to be highly palatable, appealing and energy dense with a unique combination of the main taste enhancer ingredients [122,155], generating a strong rewarding stimulus. In fact, both the rapid increase in glycaemia and vagus nerve activation, due to their composition of high refined carbohydrates and lipids composition, respectively, play an important role in triggering a dopamine release [156]. Furthermore, it has been demonstrated that brain regions involved in reward are more responsive to food stimuli with higher appetitive values than to those with lower appealing potential [137,155]. Even the shape and the appearance of UPFs are designed to preferentially activate human brain circuitries: in a study conducted by Coricelli and colleagues, 20 normal-weight adult participants viewed images of UPFs and PFs, matched for appearance, valence, arousal and, most importantly, energy density [166]. They discovered that participants were significantly faster at recognizing UPFs as foods [166] and this advantageous recognition resembles the differences observed when high-fat and low-fat foods are compared [166]. In addition, the triggering stimuli provided by UPFs align with the human tendency, especially in children who are by nature more impulsive, to prefer immediate appealing food rewards to later delivered options, even if they are larger portions [166]. Analyzing then the effect of portion sizes and energy density on the children’s brain, it seems that a reduced response in brain regions for inhibition and information processing (e.g., the prefrontal cortex) is driven by larger portion sizes, whereas a greater activation in several brain areas involved with reward and taste processing (e.g., processed in area reward, emotion control appetite regulation, and somatosensory processing) is driven by foods with higher energy density [155]. 

The discussed desirable factors of UPFs that can stimulate the reward system, the first issue to be addressed regarding children is that they are more inherently responsive to reward stimuli delivered by highly processed foods, especially in younger age [133]. Thus, excessive exposure to those foods in infancy may lastingly alter the innate hunger-satiety signals and create long-term changes to neural reward systems, promoting overconsumption [133,134]. Additional factors, also concerning children, that are responsible for the increased reward response to highly palatable foods, appear to be a primarily maladaptive eating behavior, including emotional eating, and secondarily excessive weight gain [137]. The involvement of emotional eating is understandable given the UPFs’ comfort food nature: they are consumed by the general population with the expectation of positively impacting one’s coping strategy and reducing negative emotions [164]. Effectively, the study conducted by Cummings and colleagues on young adults found that UPF consumption may be associated with a small but immediate enhancement of positive emotions (around 4–5%), and with both greater positive and lower negative emotions in the short term (1 h later) [164]. In addition, the presence of emotional eating seems to lead the subject to experience a greater mood enhancement after highly processed food intake, potentially because of the atonement to UPFs’ reinforcing effects, leading to strong anticipatory cravings, diminished control over intake, and overconsumption, followed by sustained and elevated guilt [164]. There have been no studies on children in this regard so far. Regarding higher weight status, alterations in brain regions involved in sensory processing (e.g., operculum, insular taste cortex, and orbital frontal cortex) are reported in subjects with obesity, potentially increasing sensitivity to food-related sensory stimuli, and hence to UPFs’ rewarding properties, thereby predisposing towards overconsumption [11]. One possible explanation of this enhancement in the sensory processing is the learning mechanism experienced by individuals throughout repeated exposures to UPFs, resulting in an anticipated reward response after cue external signals [87]. This increased anticipatory food reward is however combined with obesity by a blunted consummatory reward response, potentially driving to overeating as a compensatory mechanism to achieve the expected level of reward [11]. Therefore, the importance of experimentally investigating the reward system in obese children emerges. 

### 6.3. “Addictive-like Behaviour” and Ultra-Processed Foods: The Debate in the Literature on Whether or Not It Is Possible to Talk about “Addiction to Ultra-Processed Foods”

There is currently a debate in the scientific literature on whether or not it is appropriate to consider the recurrent overconsumption of UPFs as an “addiction”. 

The first school of thought (the “highly processed food addiction” perspective) suggests that UPF “addiction” may broadly mirror some psychological and behavioral aspects of substance use disorder [133,137], including [156] the high reinforcing and mood fluctuating capacity; the ability to trigger the reduced control over consumption; the strong urges or cravings; the continuous abuse despite negative consequences; and the repeated failed attempts to cut down or quit. UPFs can, in fact, trigger short-term pleasurable experiences and prompt the subject to desire to seek more, leading to a reinforcement mechanism, similar to addictive substances [164]. Similarities were also found in the brain areas activated in response to UPFs versus drug use, specifically in terms of brain regions implicated in executive functioning (e.g., attention, planning, decision-making, inhibition), reward, sensory input processing, and motor functioning [137]. Those researchers also state that, as in the case of known addictive substances, most of the consumers of UPFs do not become “addicted”: in fact, numerous individual predisposition factors (e.g., mood disorder, trauma exposure, and impulsivity) and situational factors (e.g., intake in response to negative emotions, cue-rich obesogenic environments) come into play, modulating the risk towards the development of UPF “addiction” [156]; furthermore emphasizing how the main epidemics of addictive substance use that occur are inexpensive, easily accessible, socially acceptable, and heavily marketed [156], drawing a disturbing parallel with UPFs. These researchers also underline the emerging evidence on the ability of UPFs to lead to tolerance and withdrawal [156], suggesting that children experience withdrawal when their parents restrict access to UPFs, leading them to craving, irritability, anhedonia, and negative affective symptoms, therefore predisposing to dietary change failure [133,156]. In any case, the proponents of this thesis recognize the presence of some differences from typical addictive substances, including especially the significant lower intensity of UPF withdrawal symptoms in children in respect to adult drugs withdrawal [133].

Others suggest that, considering the current sum of scientific evidence, it is excessive to define this condition as a true “addiction”. Those researchers counter argue that [156], unlike addictive substances: intravenous administration of refined carbohydrate or fat does not elicit addiction, despite the rapid availability of these nutrients to the central nervous system; UPFs do not cause a “high”; and UPFs’ activation of the reward system is weaker [156]. 

In conclusion, in light of comparing the arguments of the two streams of thoughts in the literature, “UPF addiction” remains at this time a theoretical construct with no official recognition as a diagnosable condition. It is not included as an official diagnosis in the DSM-5 (Diagnostic and Statistical Manual of Mental Disorders, 5th Edition) or the ICD-10 (International Statistical Classification of Diseases and Related Health Problems, 11th Edition) [137]; therefore, it is preferable, in the authors’ opinion, to refer to “abuse of UPFs” and not “addiction”, until irrefutable evidence is presented. In any case, despite the current absence of the attribute of “addiction”, the overconsumption of UPFs in children remains a relevant and concerning problem, not to be minimized.

## 7. Conclusions

Nutrient composition and non-nutritional properties of UPFs could explain the mechanisms underlying the relationship with obesity. UPFs are designed to be highly palatable, appealing, and energy dense with a unique combination of the main taste enhancer ingredients, generating an important rewarding stimulus, and influencing the circuits related to feeding facilitation. 

To date, food classification according to the NOVA system presents some limits. In particular, since it is a “linguistic” definition, it does not have reference cut-offs for, e.g., salt, sugar, and fat and, consequently, it is not a system capable of contributing to the overall adequacy of dietary patterns research [167]. In addition, different studies may have classified the same food as UPF or not depending on how the specific food is described in terms of ingredients and their characteristics (single ingredients vs. 2–3 vs. ≥ 5, or natural/fresh vs. imitated or industrial, and whole foods vs. fractionated substances) [116]. A lack of a clear classification system or definition of UPFs is also a limitation for efficiency in public health improvement projects [157]. In fact, UPFs are highly prevalent in the modern food environment [133], enough so that, even with their cost-effectiveness and microbiological safety, it is difficult to replace them [157]. However, there is a need for governments to take action with the aim of playing a greater role in preventive nutrition and health promotion [157]. A reduction in UPF consumption would also have implications for energy expenditure, as food processing uses significant environmental resources, such as energy, water, and packaging materials, generating much of the plastic waste stream [151]. Yet, so far, policy actions related to prevention in nutrition have prioritized interventions on the individual’s lifestyle, rather than on the commercial industry of UPFs [151]. Despite existing action plans, more ambitious food environment policies are needed [168]. Dealing with the obesity and NCD burden in European countries demands urgent implementation of supportive policies and infrastructure that enable healthy food options [168]. Prospecting potential food policy strategies to reduce the ubiquitous consumption of UPFs, it is first essential to emphasize the need for mutually reinforcing policies to drive large enough changes to change food systems and prevent NCDs [169]. The potentially complementary strategies described by the literature include fiscal policies (i.e., taxation of UPFs and subsidies or incentives to unprocessed or minimally processed foods), new front-of-pack (FOP) food labelling policies, environmental, and education based interventions (i.e., change in schools’ and communities’ food policies), public awareness media campaigns about UPFs’ negative health impacts, marketing limitations for UPFs (restrictions or bans), increasing funding for nutrition, and minimizing industry interference and influence on health policy making [162,168,169,170,171].

**Figure 5:**
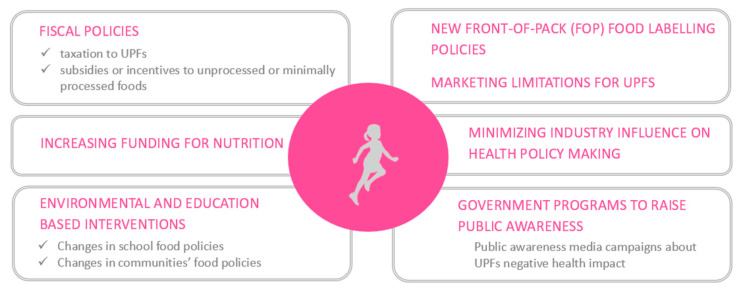
Schematic graphical representation of potential interventions applicable to counter the global overconsumption of UPFs, with a focus on the child population.

Focusing specifically on children, the school environment plays a central role in shaping eating habits and preferences as a future consumer; therefore, school food policies should provide for the removal of all UPFs from school meals and vending and for a parallel increase of real food available [162,169,170], updating school menus to offer tasty and appealing healthy recipes. In addition, schools should teach alumni the importance of following a sustainable dietary pattern, combining lessons and practical workshops, rather than continuing with the specific nutrients’ narrative (saturated fats, sodium, and sugar) [170], that has produced so far limited results. Moreover, effective food policies targeting children cannot undertake the media presence of UPF advertisements: a strategy implementable could be to apply simultaneous marketing bans on UPFs on children’s channels 24 h a day and on the general channels at specific time windows (6 a.m. to 10 p.m.) [169]. The combination of these children-focused strategies with funding initiatives for parents to choose healthier food options and to provide at home a more balanced and sustainable dietary pattern for their children is a potentially winning strategy [133]. (Figure 5) In addition, effective interventions must necessarily include a concomitant reduction in the provision of SSBs in the same settings by taking advantage of similar strategies [33,34,35,172].

Nonetheless, in order to minimize the negative outcomes related to feed intake at an early stage of development, it is important to properly understand the developmental aspects of food rewards [87]. Yet, despite the UPFs’ ubiquity within our modern food environment, knowledge on how individual UPF ingredients influence eating behavior and reward processes is lacking, especially in children. 

This review appears to be different from other reviews in the literature in several ways. Firstly, it focuses specifically on the relationship between UPFs and pediatric obesity, with a particular emphasis on the role of reward systems in regulating food intake. This is a relatively narrow focus, as many other reviews on this topic have tended to be broader in scope. Secondly, the review highlights some of the limitations of existing food classification systems, such as the NOVA system, that can affect the accuracy of studies investigating the relationship between UPFs and obesity. This is an important consideration that is not always addressed in other reviews. Thirdly, the review emphasizes the need for more ambitious food environment policies, rather than just individual-level interventions, to address the obesity epidemic. This is an important and timely call to action that is not always emphasized in other reviews on this topic. 

Further investigation of this issue could be a good starting point, along with the adoption of appropriate food and nutrition policies, to interrupt the rapid growth in the prevalence of obesity and subsequent related diseases.

## Data Availability

Not applicable.
